# A Weigh-in-Motion Characterization Algorithm for Smart Pavements Based on Conductive Cementitious Materials

**DOI:** 10.3390/s20030659

**Published:** 2020-01-24

**Authors:** Hasan Borke Birgin, Simon Laflamme, Antonella D’Alessandro, Enrique Garcia-Macias, Filippo Ubertini

**Affiliations:** 1 Department of Civil and Environmental Engineering, University of Perugia, via Goffredo Duranti 93, 06125 Perugia, Italyantonella.dalessandro@unipg.it (A.D.); egarcia28@us.es (E.G.-M.); 2 Department of Civil, Construction and Environmental Engineering, Iowa State University, Ames, IA 50011, USA

**Keywords:** smart material, smart pavement, weigh-in-motion, piezoresistive, bridge, monitoring

## Abstract

Smart materials are promising technologies for reducing the instrumentation cost required to continuously monitor road infrastructures, by transforming roadways into multifunctional elements capable of self-sensing. This study investigates a novel algorithm empowering smart pavements with weigh-in-motion (WIM) characterization capabilities. The application domain of interest is a cementitious-based smart pavement installed on a bridge over separate sections. Each section transduces axial strain provoked by the passage of a vehicle into a measurable change in electrical resistance arising from the piezoresistive effect of the smart material. The WIM characterization algorithm is as follows. First, basis signals from axles are generated from a finite element model of the structure equipped with the smart pavement and subjected to given vehicle loads. Second, the measured signal is matched by finding the number and weights of appropriate basis signals that would minimize the error between the numerical and measured signals, yielding information on the vehicle’s number of axles and weight per axle, therefore enabling vehicle classification capabilities. Third, the temporal correlation of the measured signals are compared across smart pavement sections to determine the vehicle weight. The proposed algorithm is validated numerically using three types of trucks defined by the Eurocodes. Results demonstrate the capability of the algorithm at conducting WIM characterization, even when two different trucks are driving in different directions across the same pavement sections. Then, a noise study is conducted, and the results conclude that a given smart pavement section operating with less than 5% noise on measurements could yield good WIM characterization results.

## 1. Introduction

Structural health monitoring (SHM) of road infrastructures is rapidly gaining importance in the research community. Tracking of road conditions could enable condition-based maintenance procedures, therefore resulting in important savings on a maintenance standpoint, in particular for bridges which are known to be expensive to maintain. However, conducting structural health monitoring of a bridge is difficult because of the large cost of instrumentation strategies required in covering the whole structure. A solution is to focus on clear physical quantities that can be linked to conditions. One of the key features of interest to this paper, is the identification of traffic loads, also known as weigh-in-motion (WIM) sensing. The information from WIM methods can be used directly to assess deterioration, for instance caused by over-loaded trucks [1] and by traffic usage-induced fatigue [2]. In fact, under-estimated usage rates can result in over-estimated service life [3], and, therefore, unexpected early structural failure [4]. Useful information extracted from WIM sensing includes both static and dynamic characteristics of the moving axles [5].

Widely utilized WIM sensors for pavements include bending plates [6,7,8], load cells [1,9], capacitive mats [10,11], and strip sensors [12,13], used to extract the total weight, speed, and axle count of vehicles. Bridge weigh-in-motion (BWIM) is a specialized WIM method, where loading characteristics are derived from a bridge’s response. BWIM is often conducted by employing strain gauges and the Moses algorithm that optimizes weight factors by minimizing the sum of squares of differences between measured and theoretical strains or deflections [14,15,16], and by employing accelerometers to extract information from dynamic signatures [17] using, for example, the Moving Force Identification (MFI) technique [12,18,19]. Other technologies have been proposed for WIM sensing, including contactless BWIM using two cameras to track deformation and axle spacings [20], piezoelectric sensors to measure pavement response under load [21], fiber optic sensors by measuring changes in the light beam characteristics upon load [22,23,24], and microwave-based sensors identifying changes in electric fields [25].

Despite advances in WIM sensing technologies, most of the existing solutions are expensive to deploy or intrusive. Recent technological developments in nano- and micro-technologies and digital signal processing have enabled the integration of novel sensing techniques within structural materials to create multifunctional technologies. In particular, it is now possible to produce smart pavements capable of self-sensing at costs comparable to those of regular pavements depending on the nanofiller [26]. A critical advantage of these multifunctional materials is in their high mechanical durability and environmental robustness obtained through the homogeneous integration of sensing and structural functions [27,28]. In addition, these smart materials are easier to install, more durable, require less maintenance, and depending on their fabrication process, may be less expensive to deploy than traditional sensor networks.

In this paper, a novel WIM technology based on smart pavement consisting of a piezoresistive cementitious material is investigated. The sensing principle is based on a measurable change in the electrical resistance of the material upon deformations caused by the movement of single axles. Cement-based smart materials that leverage piezoresistive effects can be fabricated, for example using carbon based conductive fillers such as carbon nanotubes (CNTs) [27] and graphite [29,30]. CNT fillers have showed good performance at the self-sensing of material deformations when deployed in a cementitious matrix [31,32,33]. The use of CNTs is also known to increase the ultimate strength and the ductility of the host material [34,35], but their environmental compatibility is not well understood [36] and result in high fabrication costs and a difficult dispersion process due to their hydrophobic characteristic [37,38]. Graphite fillers have been shown to be a good alternative due to their lower costs and easier dispersion relative to CNTs, but with the trade-off of a lower sensitivity to strain [38,39].

A challenge in using these piezoresistive materials is in mapping the electrical signal to WIM characteristics. Typically, the desired characteristics of information from a WIM system are axle weights, axle separation, and vehicle speed [40]. While the passage of an axle can be easily detected through the inspection of high frequency components of a given signal [41], the determination of axle weights is complex because of the non-linearity of the data [42,43] and requires the construction of a representation. Data-based techniques are popular for such task, because they do not require physical knowledge. However, they yield a black-box representation with no physical meaning. Data-based techniques applied to WIM sensing include the use of neural networks [44] and principal component analysis [45] to classify vehicles from strain time histories, and decision trees and k-means clustering [46] or neural networks [47,48] to conduct WIM from inductive signature data [49].

In this paper, we propose and investigate an algorithm enabling WIM sensing based on signals acquired from piezoresistive pavement sections. The algorithm starts by generating basis signals from axles using a finite element model of the structure equipped with the smart pavement and subjected to given vehicle loads. Subsequently, the measured signal is matched by finding the number and weights of appropriate basis signals that would minimize the error between the numerical and measured signals, yielding information on the vehicle’s number of axles and weight per axle, therefore enabling vehicle classification capabilities. Then, the temporal correlations of the measured signals are compared across smart pavement sections to determine the vehicle weight.

The rest of the paper is organized as follows. Section 2 describes the pavement-based WIM system. Section 3 investigates the algorithm for WIM characterization on the smart pavements. Section 4 presents results from the numerical simulations.

## 2. Smart Pavement-Based Weigh-In-Motion System

**Figure 1:**
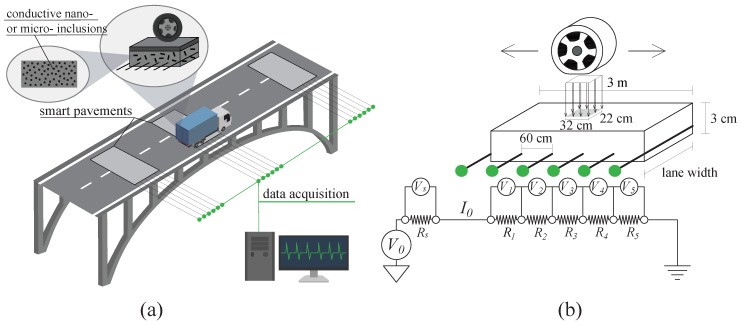
Smart pavement-based weigh-in-motion (WIM) system illustrating (**a**) the integrated smart pavement within the bridge system; and (**b**) the electrical principle.

The integration of the smart composites in a bridge system can be conducted either in the form of continuous or discontinuous (i.e., sections) layers. Here, distinct smart pavement sections are used to facilitate signal separation from different measurement points, as illustrated in Figure 1a showing an axle moving over a section of the smart pavement. Smart pavement sections are envisioned to be approximately 3 m long (along traffic) spanning the width of traffic lanes, with the smart materials located under the asphalt layer (when asphalt is used). The sensing layout illustrated in Figure 1b shows six electrodes distributed along each sensing pavement section. From the EN-1991-2 [50], assuming a typical wheel contact area of 32 cm in length and 22 cm in width, an electrode separation of 60 cm would allow to capture full wheel characteristics between electrodes to measure the axle weight more accurate. [41]. In this configuration, at least three distinct smart pavement sections must to be placed along the road bridge in order to estimate the speed of the vehicle to allow for the determination of axle separation through spatio-temporal comparisons and cross-validation of data.

### Measurement Principle

**Figure 2:**
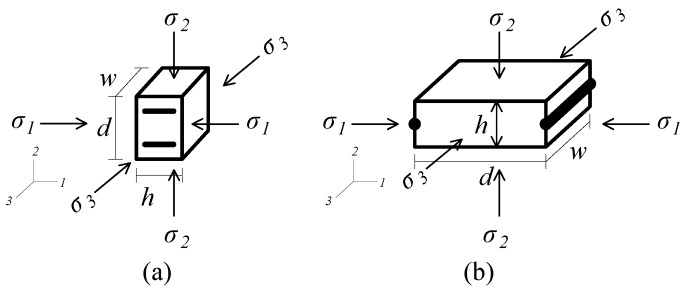
Illustration of the piezoresistive material with two different electrode configuration showing the orientation of mechanical stresses σ: (**a**) electrode setup adapted from [51]; and (**b**) smart pavement segment between two consecutive electrodes.

The smart pavement simulated in this section has an electromechanical behavior inspired by research on graphene-cement composites in [51]. The measurement principle of the smart pavement consists of mapping the electrical signal to a change in deformation provoked, for instance, by the passage of an axle, through an electromechanical model. To derive the electromechanical model, we start from the configuration used in [51] and illustrated in Figure 2a to link their experimental results (i.e., gauge factor) to our proposed configuration illustrated in Figure 2b. The configuration shown in Figure 2a has electrodes separated by a distance *d*, and an overall width *w* and height *h*. The load is applied directly along axis 2. Mechanical stresses are denoted $\sigma_{1,2,3}$, where the subscript denotes the axis direction. The resistance *R* of the material measured from the electrodes can be written:

(1) $$R=\rho \frac{d}{h\cdot w}$$

where ρ is the resistivity of the material, *d* is the distance between electrodes and h·w is the value of cross-sectional area of the segment. The change in the resistance can be formulated as follows assuming an isotropic material:

(2) $$\begin{array}{cc} \frac{\Delta R}{R} & =\frac{\Delta \rho }{\rho }+\frac{\Delta d}{d}-\frac{\Delta h}{h}-\frac{\Delta w}{w} \end{array}$$

(3) $$\begin{array}{cc} \; & =\frac{\Delta \rho }{\rho }+\left(1+2\nu \right)\varepsilon_{1}-\varepsilon_{2}-\varepsilon_{3} \end{array}$$

where ν is the Poisson’s ratio and ε denotes strain along the axis associated with the subscript. Equation (3) can be re-written using $\varepsilon_{1}=\varepsilon_{3}=-\nu \varepsilon_{2}$ assuming pure axial loading along axis 2:

(4) $$\frac{\Delta R}{R}=\frac{\Delta \rho }{\rho }-\left(1+2\nu^{2}\right)\varepsilon_{2}$$

Note that the term Δρ/ρ denotes the piezoresistive effect of the material, which is typically evaluated experimentally through the estimation of the gauge factor λ:

(5) $$\lambda =-\frac{\frac{\Delta R}{R}}{\varepsilon_{1}}=-\frac{\frac{\Delta \rho }{\rho }-\left(1+2\nu^{2}\right)\varepsilon_{2}}{\varepsilon_{1}}$$

Ref. [51] found a range λ=258 to 4139 for cement composites. The value λ=258 was adopted and used for the numerical simulations. Remark that with the smart pavement configuration (Figure 2), the strain of interest is along direction 2 ($\varepsilon_{2}$), and Equation (5) is modified using $\varepsilon_{1}=-\nu \varepsilon_{2}$:

(6) $$\nu \lambda =\frac{\frac{\Delta \rho }{\rho }}{\varepsilon_{2}}-\left(1+2\nu^{2}\right)$$

where the actual gauge factor in the simulations is taken from [51] (λ=258), but pre-multiplied by ν. The selection of λ influences the signal-to-noise ratio, where the signal noise decreases with increasing λ. Here, a lower bound on λ is selected to enable a conservative evaluation of the algorithm performance.

A smart pavement layer consists of six electrodes and can be modeled as a series of resistors, as illustrated in Figure 1b. Upon the movement of a force over the smart section, the composite deforms and provokes a change in resistance $R_{i}$ for i=1,2,…,5. A change in resistance is measured through a measurable change in potential $V_{0}$ between the first and last electrodes, where the last electrode is grounded as shown in Figure 1b. A typical value for $V_{0}$ is 30 V. A linear distribution of voltage difference among electrodes is expected without the existence of the mechanical strain, because $R_{1}=R_{2}=R_{3}=R_{4}=R_{5}$ and current $I_{0}$ is constant through the circuit, under the assumption of perfect contact conditions between electrodes and the smart material. In order to calculate $R_{1}$ through $R_{5}$, five voltage drops are measured, the voltage drop $V_{s}$ along the shunt resistor $R_{s}$ is recorded and $I_{0}$ is determined using Ohm’s Law:

(7) $$I_{0}=\frac{V_{s}}{R_{s}}$$

**Figure 3:**
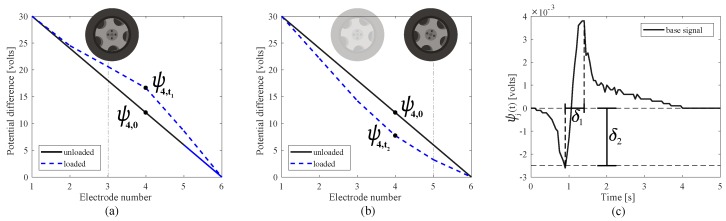
(**a**) The unloaded and loaded (at time *t*) voltage distributions across electrodes (**a**) for the axle located between the 1st and 4th electrodes; and (**b**) for the axle located between the 4th and 6th electrodes. (**c**) Typical basis signal ψ(t) obtained from the passing of an axle.

The computed variations from the initial voltage distribution is the signal used in conducting the WIM identification, and its time series measurement collected during the passage of a vehicle is denoted as Ψ(t). This is done through the algorithm explained in the upcoming subsection. Note that in this study, we use the signal from two segments: one is the segment located between the first and the forth electrode, and the other is the segment located between the fourth and last electrodes (see Figure 3a–c). The selection of these sections were based on a signal sensitivity analysis conducted using a finite element model. While only two segments are considered in our study, having distributed electrodes within the pavement enhances the flexibility of the detection system for different types of tires, types of vehicles, and speeds. This is left to future work.

## 3. Algorithm for WIM Characterization

The WIM identification algorithm begins with the assumption that the measured potential difference $\Psi \left(t\right)=V_{0}\left(t\right)-V_{k}\left(t\right)$, where *k* is the index of electrode according to Figure 1b, in response to a moving vehicle can be represented as a linear superposition of responses from the *n* individual axles $\psi_{j}\left(t\right)$:

(8) $$\Psi \left(t\right)=\sum_{j=1}^{n}\psi_{j}\left(t\right)$$

where t=0 is taken as the time of the axle moving over the first electrode. Axle responses $\psi_{j}\left(t\right)$ are generated using a physical representation of the system, here a finite element model (FEM). This is done by moving a single axle of known weight along the bridge and simulating the voltage readings at each electrode. The voltage difference time history is obtained after subtracting the mean voltage reading of the unloaded state.

To illustrate, example voltage readings $\psi \left(t\right)=V_{0}\left(t\right)-V_{4}\left(t\right)$ (see Figure 1b) are shown in Figure 3 from a simulated moving axle. Figure 3a plots the signal for the axle located between electrodes 1 and 4, and Figure 3b for the axle located between electrodes 4 and 6, with the vertical lines showing position of the axle and electrode 4. In this example, basis signal $\psi_{j}\left(t\right)$ is taken as $\psi_{4,0}-\psi_{4,t}$, where $\psi_{4,0}$ denotes the unloaded state voltage reading at the fourth electrode and $\psi_{4,t}$ denotes the loaded voltage reading at the fourth electrode at time *t*. Figure 3c plots a typical time history ψ(t) produced by simulating a moving load of 90 kN on the FEM at a constant speed of 10 m/s, where chattering in the curve is a feature of the FEM’s mesh size. Signal ψ(t) is characterized by a peak-to-peak temporal distance ($\delta_{1}$) and a maximum negative magnitude ($\delta_{2}$), which maps to the axle distance through vehicle speed and weight of the axle, respectively, with $\delta_{1}$ decreasing with increasing speed, and $\delta_{2}$ increasing with increasing weight. Note that ψ(t) is also a waveform starting and ending at zero (i.e., unloaded state).

**Figure 4:**
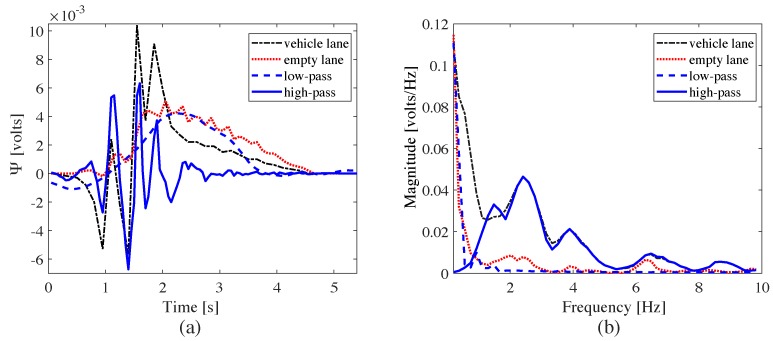
Comparison of low and high frequency components of a typical signal: (**a**) time domain; and (**b**) frequency domain.

In field implementations, signal ψ(t) comprises the response of the pavement to both the structure and axle movements. These components characterize the low and high frequency contents of the signal, respectively. This separation of signals is conducted through a discrete wavelet transform (DWT). For this study, the cut-off frequency is set to 1.25 Hz, selected by simulating the response of the modeled bridge and comparing the smart pavement responses between the empty (i.e., no vehicle) and vehicle (i.e, occupied) lanes. Figure 4 illustrates the signal separation task through a typical time series and frequency measured responses from the passage of a vehicle. Figure 4a plots signal Ψ(t) measured from the vehicle lane, from the empty lane, the low-pass filtered measured response, and the high-pass filtered measured response. One can observe that the low-pass filtered signal mimics that of the empty lane response in both the time and frequency domains. It follows that only the high frequency content of vehicle lane signal Ψ(t), denoted $\Psi^{H}\left(t\right)$, is used for the WIM characterization, and Equation (8) becomes

(9) $$\Psi^{H}\left(t\right)≅\sum_{j=1}^{n}\psi_{j}^{H}\left(t\right)$$

where $\psi_{j}^{H}\left(t\right)$ is the high frequency component of the *j*th basis signal. The determination of the basis signals used in reconstructing $\Psi^{H}\left(t\right)$ is conducted through the following search procedure. First, the maximum negative magnitudes of the measured and basis signals are normalized to $\delta_{2}=-1$. Second, the location of the negative magnitudes between the measured and basis signals is matched by shifting the basis signal by $H_{j}$, where $H_{j}$ is a square matrix time shift operator, and the basis signal that reduces the L2-norm is selected. Third, the matched basis signal is subtracted from the measured signal, the L2-norm of the remaining signal is recorded, and the operation repeated to find the other basis signals. The process is iterated until an increase in the recorded L2-norm is observed due to redundant subtractions. Fourth, scale factors $a_{j}$ that minimize the error function *E* are computed:

(10) $$E={∥\Psi^{H}\left(t\right)-\sum_{j=1}^{n}a_{j}H_{j}\psi_{j}^{H}\left(t\right)∥}^{2}$$

where $H_{j}$ can be mapped to axle spacing once the vehicle speed is determined, and scale factors $a_{j}$ can be mapped to axle and vehicle weights once the relationship between the static and dynamic weights are established using the FEM. Note that Equation (9) is Equation (10) specialized with $a_{j}$ and $H_{j}$ being unitory. The minimization of Equation (10) is conducted using the extremum seeking algorithm by Krstic [52]. We selected this more general solver due to its capacity to handle nonlinear objective functions that are anticipated in future developments of the algorithm. The principle of the algorithm is to have the objective function approach and oscillate around its extremum value, and let the variables undergo high frequency changes to scan for optimal values in the neighborhood. First, estimated parameters $a_{j}$ are sequentially updated using following update rule from [52]:

(11) $${\dot{\hat{a}}}_{j}=-k(\dot{E}\left({\hat{a}}_{j}\right)-g{\tilde{y}}_{o})\sin\left(\omega \tau \right)$$

where *k* and *g* are the adaptation gain and cut-off frequency, respectively, ω is the frequency parameter and usually selected such that ω>g, and τ denotes a discrete time step in the optimization space. Variable ${\tilde{y}}_{o}$ is taken as

(12) $$\begin{array}{ccc} {\tilde{y}}_{o}^{\tau +\Delta \tau } & = & {\tilde{y}}_{o}^{\tau }+{\dot{\tilde{y}}}_{o}\Delta \tau \end{array}$$

(13) $$\begin{array}{ccc} {\dot{\tilde{y}}}_{o} & = & \dot{E}\left({\hat{a}}_{j}\right) \end{array}$$

and $\dot{E}\left({\hat{a}}_{j}\right)$ is calculated by finite difference:

(14) $$\dot{E}\left({\hat{a}}_{j}\right)=\frac{E\left({\hat{a}}_{j}^{\tau +\Delta \tau }\right)-E\left({\hat{a}}_{j}^{\tau }\right)}{\Delta \tau }$$

and it follows that

(15) $${\hat{a}}_{j}^{\tau +\Delta \tau }={\hat{a}}_{j}^{\tau }+\alpha \sin\left(w(\tau +\Delta \tau )\right)+{\dot{\hat{a}}}_{j}\Delta \tau$$

where α can be considered as an exploration range of the optimization space. At initialization, ${\tilde{y}}_{o}^{\tau }$ can be selected as 0 for τ=0. Using the extremum seeking algorithm, $|a_{j}-{\hat{a}}_{j}|\to 0$ as τ→∞ and $E({\hat{a}}_{j})$ converges to its extremum value and floats around extremum. The error function *E*, will converge to 0. Figure 5 illustrates an example of the basis signal search procedure using a 2-axle vehicle weighting 50 kN each and separated by 6 m. Figure 5a,b plots the sequential identification of the two basis signals, showing the respective signal matches with the measured signal. Figure 5c visualizes the time shift operators $H_{j}$ and scale factors $a_{j}$. Figure 5d plots the resulting signal match after Equation (10) is minimized. Speed is determined by temporally correlating signals across smart pavement sections. The use of more than two sections allows for an increase in WIM accuracy.

For a linear elastic model and this study, the sum of the coefficients *a* is the weight of the vehicle. In the field a map between coefficients and actual weight is required, which can be achieved with a calibration procedure.

In summary, the WIM algorithm is conducted through the following sequential steps:

1. Identification of weights, time shifts, and number of axles from the signal collected in the initial pavement section by minimizing Equation (10);
2. Reconstruction of the signal (Equation (9)) from the initial pavement section;
3. Temporal identification of vehicle through the computation of correlation between the reconstructed signal and the measured signal from the subsequent pavement sections; and
4. Determination of vehicle speed through the averaging of temporal identification data.

## 4. Numerical Simulations

### 4.1. Numerical Model

**Figure 6:**
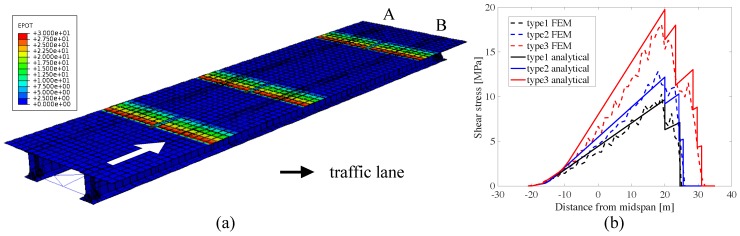
(**a**) Finite element model (FEM) of the bridge showing the change in potential along smart pavement sections, where smart pavement sections are under a charge of 30V, and (**b**) analytical versus numerical influence lines for the Von Mises Stress at the end of the bridge.

The proposed smart pavement-based WIM characterization is simulated on the numerical model of an existing structure consisting of the approach bridge to the arch bridge in Valnerina, Terni, Italy. This approach bridge is a steel-concrete composite structure with a 40 m-span length of 12 m width. The FEM model, shown in Figure 6, is constructed and simulated in ABAQUS [53]. It is created by solid elements. The first three natural frequencies of the simulated system are 2.78 Hz (bending), 4.61 Hz (torsional), 8.06 Hz (bending). The smart pavement sections from the FEM are simulated with six line-electrodes each, spaced evenly (Figure 1b). They are built in the FEM through coupled thermal-electrical elements of user-defined conductivity. The piezoresistivity of the material is induced through a subroutine of user-defined elements built from Equation (6) [54].

Numerical simulations are conducted using three types of trucks passing on the right lane of the bridge (Lane-B in Figure 6a). Initial simulations run the trucks one by one at a speed of 10 m/s (36 km/h). These trucks are defined in Eurocode 1 [50] and the information is presented in Table 2. The real distances between axles are given in the “simulation input” column. The total weights of the trucks are defined as 280 kN, 360 kN and 630 kN for type 1, 2 and 3 respectively. The simulation is run to show that vehicle characteristics can be determined in the presence of the structural deformation signal. A case is simulated with two trucks running on the each lane simultaneously, in opposite directions, in order to verify the robustness of the algorithm. For this case, a type 2 truck is ran on the right lane with the speed of 10 m/s (36 km/h), while a type 1 truck is ran on the left lane with the speed of 15 m/s (54 km/h). The time step for the simulation is selected as 0.025 s to capture the signal of the faster vehicle located on the left lane. The aim of the second simulation is to show that the proposed algorithm is capable of acquiring different speeds and the vehicle characteristics despite the presence of other vehicles on the road. The speeds under investigation in this study are on the low-end of the average speed of a vehicle in order to reduce computational demand on the numerical model. Nevertheless, it is expected that results would extend to different speed cases. This is to be validated in future work involving field experiments. That study is executed with a 1% Gaussian noise added to the signals. A third set of simulations is conducted to study the effect of noise on the algorithm. The simulations consist of adding various noise levels to the signal produced by all three types of trucks and assessing the WIM characterization capabilities.

Numerical outputs from the first set of simulations are used to validate the model. Analytical and numerical shear force influence lines taken at the end support of the section B girder are compared. Figure 6b plots the influence lines for each truck. As plotted in the figure, the expected step changes due to separation of the axles can be observed very significantly with the FEM. The values are consistent with the analytical calculations using tha equivalent beam model and the observed peaks are due to the incompatibility of moving load with constant speed and the mesh of the FEM.

### 4.2. Results and Discussion

#### 4.2.1. Results of WIM

**Figure 7:**
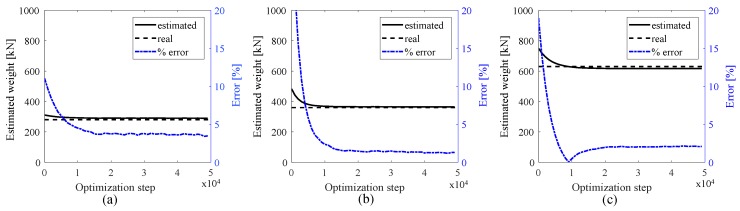
Weight estimations for the first simulation case; (**a**) type 1 truck; (**b**) type 2 truck; and (**c**) type 3 truck.

The first investigation consists of evaluating the capability of the algorithm at estimating the weight for all three types of trucks using the signal of the first smart pavement section. Figure 7 plots the results, showing a convergence of the algorithm as the optimization progresses. The residual error, below 5% in every case, is attributed to the spatiotemporal resolution of the numerical simulation. The number of axles for type 1, type 2, and type 3 trucks were correctly identified as 2, 3, and 5 axles, respectively. Results from the WIM algorithm are listed in Table 2. Note that because this study only considers three different trucks, vehicle classification is a simple task given that it can be conducted through a simple axle count. In the event of a different type of vehicle or an overweight truck, provided that the correct basis functions exist for an accurate WIM identification, it would be up to a classification algorithm to decide in which category the truck would fall using the WIM identification data from the proposed algorithm. This is left to future work.

The weight and axle data are used to reconstruct the signals ${\hat{\Psi }}^{H}$, where the hat denotes an estimation, for all of the pavement sections using Equation (9), including that from the initial section (P1), giving raise to ${\hat{\Psi }}_{1}^{H}$, ${\hat{\Psi }}_{2}^{H}$, and ${\hat{\Psi }}_{3}^{H}$ for sections P1, P2, and P3 (as illustrated in Figure 6a), respectively. These estimated signals can be seen as a pattern produced by the vehicle that should be recognized in order to identify such vehicle when driving over a given pavement section. This pattern recognition task is conducted by comparing the correlations between the estimated (i.e., reconstructed) and measured signal $\Psi^{H}$. Table 1 lists the cross-correlation results normalized to unity for the highest correlation. The cross-correlations between the estimated and measured signal at the same locations (i.e., diagonal values) have values of 1.0, therefore demonstrating success of pattern recognition. Remark that the cross-correlation values ${\hat{\Psi }}_{1}^{H}$-$\Psi_{2}^{H}$ for all three types of trucks are higher than 0.90, which indicates possible error in the recognition tasks under higher levels of noise. This will be investigated later under simulation case 3.

Subsequently, the truck signal is identified across pavement sections, the speed is determined by averaging the travel time over the distance between sections P1-P2 and P2-P3. The obtained speeds were the same for all three truck types, with 9.1 m/s and 10.7 m/s between sections P1-P2 and P2-P3, respectively, yielding a mean speed of 9.9 m/s. This is a 1% error compared with the simulated speeds of 10 m/s, this can be attributed to the discrete simulation time step of 0.05 s.

**Figure 8:**
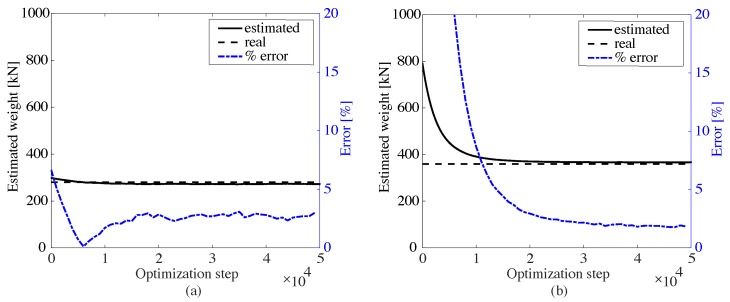
Weight estimations for the second simulation case; (**a**) type 1 truck; (**b**) type 2 truck.

The same optimization procedure is conducted for the second simulation case. The base signals from the first investigation are used for WIM characterization using the right lane signal, while new base signals are produced for WIM characterization using the left lane signal. Figure 8 shows the weight estimation results, with the identified weights and axle distances listed in Table 2. A residual error below 5% is also observed. One can also observe that the error exhibits chattering, which is attributed to the presence of a vehicle in the other lane. Here, the computed mean speeds were 14.8 m/s and 9.9 m/s for truck types 1 and 2, respectively. Compared with the simulated speeds of 15 m/s and 10 m/s, the estimation errors are within 1% for both types of truck, again due to the discrete time steps used in the numerical simulations.

#### 4.2.2. Noise Study

In this subsection, a noise study is conducted to evaluate the robustness of the algorithm. The study consists of conducting WIM characterization on the three truck types, passed on the bridge individually, under various levels of noise. Noise is modeled as a zero-mean Gaussian process with standard deviation $\sigma_{N}=\alpha \max\left(|\Psi \left(t\right)|\right)$ where α is a number between 0 and 1. Values for α=0,1,2,5,10,15,20,25% are investigated, with 30 realizations produced for each α.

**Figure 9:**
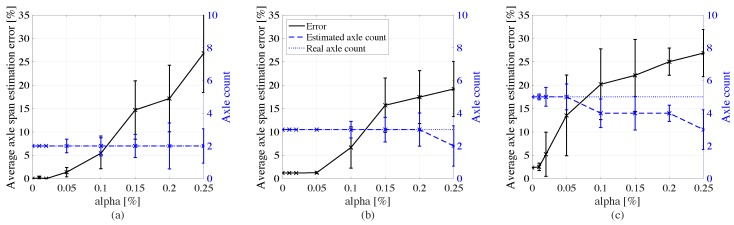
Axle span-to-vehicle length estimation errors (with +/- standard deviation intervals) as functions of noise level for: (**a**) type 1 truck; (**b**) type 2 truck; and (**c**) type 3 truck.

Figure 9 plots results on the axle characterization for each type of truck. The average axle count is rounded to the nearest integer. Acceptable estimation on the axle distances are achieved under noise levels of 5%, where the error stays below 5% of the total length between axles of the vehicle for truck types 1 and 2, and below 10% for truck type 3, yet returning the correct axle count. Beyond 5% noise, axles may be miscounted, as made more evident under truck type 3 likely due to the higher complexity of the signal arising from the higher number of axles. This would lead to incorrect WIM characterization.

**Figure 10:**
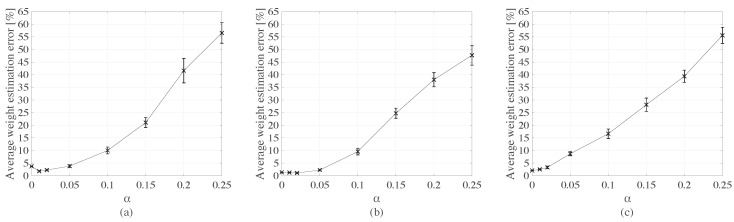
Mean weight estimation error (with +/- standard deviation intervals) as functions of noise level for: (**a**) type 1 truck; (**b**) type 2 truck; and (**c**) type 3 truck.

Figure 10 plots the mean weight estimation error results as functions of α for each type of truck. The estimation error remains under 10% for all three types of trucks for noise levels below 5%, after which the error and variance increase significantly with increasing noise.

**Figure 11:**
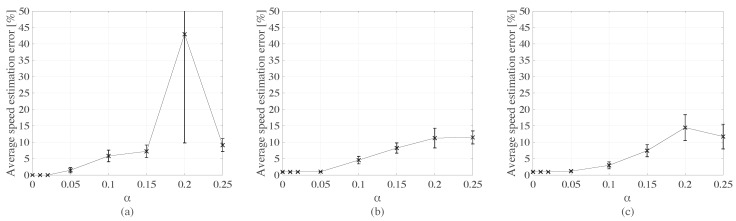
Mean speed estimation error (with +/- standard deviation intervals) as functions of noise level for: (**a**) type 1 truck; (**b**) type 2 truck; and (**c**) type 3 truck.

Figure 11 plots the mean speed estimation error as a function of α for each type of truck. It is observed that the estimated speed error is low and constant for all types of trucks and starts increasing approximately passed the 5% mark. There is also a mischaracterization of vehicle signals that occurs under high noise, as hypothesized earlier. This is made evident in Table 3 summarizing signal cross-correlation results under three distinct levels of noise: α= 2.5%, 10%, and 20%. It can be observed, for example, that the cross-correlation of signals ${\hat{\Psi }}_{P1}^{H}$ and $\Psi_{P2}^{H}$ for truck type 1 augments with noise until it is misidentified under α = 20%. This metric decreases between α= 10% and α = 20%, as it is also evident in Figure 11. For the other truck types, this can be attributed to the richer signals caused by a higher number of axles.

From the noise investigation, it can be concluded that a smart pavement system yielding accurate WIM characterization performance is one that yields measurements with a noise level below 5% of the peak signal value. Under that level, the proposed algorithm performs well at characterizing the number of axles, vehicle weight, and vehicle speed.

## 5. Conclusions

In this study, a novel algorithm for weigh-in-motion characterizing leveraging signals from smart, self-sensing composite pavements was proposed. The algorithm starts by applying a high-pass filter on the signal collected from the smart pavement, and multi-variable optimization is conducted to match the signal using a pre-produced bank of basis signals. This yields information on the weight and number of axles. Then, the signal is reconstructed using the identified weights and basis signals, and temporal cross-correlations of signals across smart pavement sections studied in order to identify vehicle speed.

The proposed algorithm was verified through numerical simulations consisting of passing of three different types of truck, as defined by the Eurocode, over a modeled bridge. Trucks were passed non-simultaneously at a constant speed of 10 m/s, and then two different trucks were passed simultaneously in opposite directions at constant speeds with one at 10 m/s and the other at 15 m/s. Lastly, the robustness of the algorithm was evaluated by adding various levels of Gaussian noise to the synthetic signals.

Results from the numerical simulations showed that the algorithm was capable of appropriately conducting WIM characterization of vehicles, even when two different trucks were passed simultaneously and in opposite directions. In the presence of noise in the collected signals, the algorithm performed well at the WIM characterization task for noise levels under 5% of the peak signal amplitude, therefore setting a performance target for the development of self-sensing materials for smart pavements.

Overall, work presented in this paper laid algorithmic foundations for WIM sensing using smart pavements, de facto enabling the integration of smart materials within our transportation infrastructure. Future work will include more diverse numbers and types of vehicles, as well as laboratory and field validation.

## Figures and Tables

**Figure 5:**
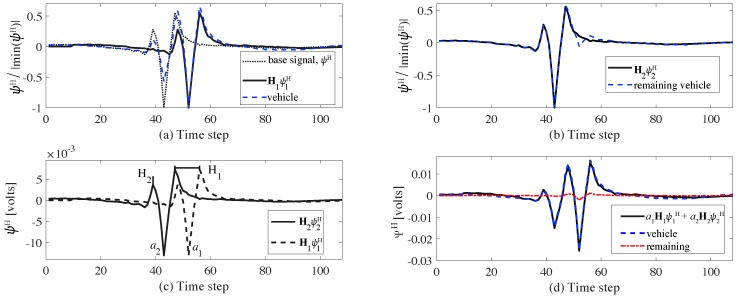
Basis signal search procedure: (**a**) normalization and matching of the signals to find the first basis signal $H_{1}\psi_{1}^{H}$; (**b**) matching of remaining signal to find the second basis signal $H_{2}\psi_{2}^{H}$; (**c**) visualization of time shift operators $H_{1}$ and $H_{2}$, and scale factors $a_{1}$ and $a_{2}$; and (**d**) reconstructed signal.

**Table 1:** Cross-correlation tables of the estimated signals (${\hat{\Psi }}^{H}$) against measured signals ($\Psi^{H}$) from each pavement section (P1 to P3) under the first simulation case for each type of truck:

Type 1	$\Psi_{P1}^{H}$	$\Psi_{P2}^{H}$	$\Psi_{P3}^{H}$	Type 2	$\Psi_{P1}^{H}$	$\Psi_{P2}^{H}$	$\Psi_{P3}^{H}$	Type 3	$\Psi_{P1}^{H}$	$\Psi_{P2}^{H}$	$\Psi_{P3}^{H}$
${\hat{\Psi }}_{P1}^{H}$	1.00	0.91	0.48	${\hat{\Psi }}_{P1}^{H}$	1.00	0.95	0.53	${\hat{\Psi }}_{P1}^{H}$	1.00	0.97	0.35
${\hat{\Psi }}_{P2}^{H}$	0.74	1.00	0.63	${\hat{\Psi }}_{P2}^{H}$	0.73	1.00	0.70	${\hat{\Psi }}_{P2}^{H}$	0.69	1.00	0.70
${\hat{\Psi }}_{P3}^{H}$	0.43	0.54	1.00	${\hat{\Psi }}_{P3}^{H}$	0.66	0.57	1.00	${\hat{\Psi }}_{P3}^{H}$	0.25	0.44	1.00

**Table 2:** WIM simulation inputs and outputs, listing results for the estimation of axle spacings (axle sp) and axle weights (axle w), along with the percentage estimation error (err)).

	Simulation Input		Simulation Output							
	Eurocode		simulation case 1				simulation case 2			
	axle sp	axle w	axle sp	err	axle w	err	axle sp	err	axle w	err
	(m)	(kN)	(m)	(%)	(kN)	(%)	(m)	(%)	(kN)	(%)
type 1	4.5	90	4.5	0	90	0	4.5	0	81	10
		190			196	3			177	7
type 2	4.2	80	4	4	77	4	4.5	7	93	16
	1.3	140	1.5	15	125	10	1	23	162	15
		140			155	10			115	17
type 3	3.2	90	3	6	91	1				
	5.2	180	5.5	6	171	5				
	1.3	120	2	53	148	23				
	1.3	120	1.5	15	158	32				
		120			46	62				

**Table 3:** Cross-correlation tables of the estimated signals (${\hat{\Psi }}^{H}$) against measured signals ($\Psi^{H}$) from each pavement section (P1 to P3) for the three types of trucks under α= 2.5%, 10%, and 20% levels of noise.

		α=2.5%			α=10%			α=20%		
		$\Psi_{P1}^{H}$	$\Psi_{P2}^{H}$	$\Psi_{P3}^{H}$	$\Psi_{P1}^{H}$	$\Psi_{P2}^{H}$	$\Psi_{P3}^{H}$	$\Psi_{P1}^{H}$	$\Psi_{P2}^{H}$	$\Psi_{P3}^{H}$
	${\hat{\Psi }}_{P1}^{H}$	1.00	0.91	0.48	1.00	0.96	0.46	0.73	1.00	0.91
Type 1	${\hat{\Psi }}_{P2}^{H}$	0.72	1.00	0.66	0.73	1.00	0.48	1.00	0.75	0.65
	${\hat{\Psi }}_{P3}^{H}$	0.42	0.50	1.00	0.31	0.47	1.00	0.40	0.47	1.00
	${\hat{\Psi }}_{P1}^{H}$	1.00	0.95	0.53	1.00	0.99	0.53	1.00	0.90	0.44
Type 2	${\hat{\Psi }}_{P2}^{H}$	0.73	1.00	0.70	0.75	1.00	0.68	0.80	1.00	0.94
	${\hat{\Psi }}_{P3}^{H}$	0.66	0.57	1.00	0.57	0.60	1.00	0.49	0.61	1.00
	${\hat{\Psi }}_{P1}^{H}$	1.00	0.97	0.35	1.00	0.98	0.43	1.00	0.78	0.12
Type 3	${\hat{\Psi }}_{P2}^{H}$	0.69	1.00	0.70	0.78	1.00	0.64	0.86	1.00	0.26
	${\hat{\Psi }}_{P3}^{H}$	0.25	0.44	1.00	0.19	0.42	1.00	0.31	0.28	1.00
